# Public Awareness of Diet-Related Diseases and Dietary Risk Factors: A 2022 Nationwide Cross-Sectional Survey among Adults in Poland

**DOI:** 10.3390/nu14163285

**Published:** 2022-08-11

**Authors:** Adam Żarnowski, Mateusz Jankowski, Mariusz Gujski

**Affiliations:** 1 Department of Public Health, Medical University of Warsaw, 02-097 Warsaw, Poland; 2 School of Public Health, Centre of Postgraduate Medical Education, 01-826 Warsaw, Poland

**Keywords:** diet, diseases, diet-related diseases, dietary patterns, dietary risk factors, health, Poland

## Abstract

A suboptimal diet is a risk factor for numerous non-communicable diseases. This study aimed to assess the level of knowledge on diet-related diseases and dietary risk factors among adults in Poland as well as to identify factors associated with awareness of diet-related diseases and dietary risk factors. This cross-sectional survey was carried out in July 2022 on a representative sample of adults in Poland. Data were received from 1070 individuals (53.3% females) aged 18–89 years. Out of eight diet-related diseases included in this study, overweight/obesity was the most recognized diet-related disease (85.0%). Stroke (26.2%) and osteoporosis (17.9%) were the least recognized diet-related diseases. Out of eight dietary risk factors included in this study, excessive consumption of sugar and salt (73.4%) was the most recognized dietary risk factor. Less than half of the respondents were aware that (1) too little vitamin intake, (2) too little intake of calcium and magnesium, (3) too little consumption of fish and oils, and (4) too little dietary fiber intake can lead to the development of the diseases. Having higher education and the presence of chronic diseases were the most important factors associated with a higher level of awareness of diet-related diseases and dietary risk factors (*p* < 0.05).

## 1. Introduction

A suboptimal diet is an important preventable risk factor for numerous non-communicable diseases (NCDs) [1,2,3]. It is estimated that in 2017, approximately 11 million deaths worldwide were attributed to dietary risk factors [3]. Diet-related NCDs include overweight/obesity, cardiovascular diseases (such as arterial hypertension, myocardial infarction, stroke), diabetes mellitus, certain cancers, and osteoporosis [4]. An unhealthy diet also significantly contributes to the development of a cluster of disorders known as metabolic syndrome [5]. Diet-related NCD burden is expected to increase with population aging and increasing obesity rate in numerous countries [6]. 

There are numerous dietary risk factors linked to the development of diseases [7,8]. However, excessive sodium intake, low intake of whole grains, as well as low intake of fruits are considered the most important dietary risk factors [3]. Moreover, excessive consumption of saturated and trans fats also contributes to cardiovascular mortality [9]. Another important risk factor is excessive free sugar intake, which increases the risk for tooth decay, obesity, and cardiovascular diseases [10]. 

National consumption of major food groups differs across countries [6]. Diet quality varies by gender, age, and socioeconomic status [11]. Moreover, the global nutrition transition also has a significant impact on the dietary habits of populations [12,13]. Rapid urbanization, industrialization, and changing lifestyles have led to shifts in dietary patterns, especially in developing countries [12,14]. As the result of the global nutrition transition, an increase in consumption of processed foods, sugar-sweetened beverages, calorific and fatty food intake, and eating out, as well as an increase in food portion sizes, was observed [14,15]. At the same time, a lower intake of fruit, vegetables, and high-fiber foods/whole grains was noted [14,15,16].

Individual dietary behaviors and nutrient intake also depend on nutrition knowledge [17,18]. Promoting healthy eating is one of the major goals of public health [14,19]. High public awareness of a healthy diet and nutrition is crucial to limit the burden of diet-related NCDs [20]. However, there is a limited number of scientific data on public awareness of diet-related diseases and dietary risk factors. Moreover, factors associated with public awareness of diet-related diseases are poorly understood.

Poland is a high-income country in Central and Eastern Europe (CEE) that has undergone a substantial transition over the past three decades [21]. After communism collapsed in 1989 and Poland joined the European Union (EU) in 2004, the food market in Poland changed rapidly [21,22]. An increase in the gross domestic product (GDP), urbanization, and changes in Poland’s agricultural sector had a significant impact on the dietary behaviors of the inhabitants of Poland [22]. Changes in nutritional behaviors led to an increase in the prevalence of overweight or obesity among adults in Poland [22,23]. The portion of overweight adults in Poland is higher than the EU average (58% vs. 53%) [23]. According to the National Institute of Public Health—National Institute of Hygiene estimates, approximately 10 million Poles have arterial hypertension, over 3.1 million suffer from diabetes mellitus, and approximately 2.5 million females and 500 thousand males have osteoporosis [24]. Moreover, every year over 150,000 new cancer cases are detected and over 100,000 new cases of myocardial infarction are reported in Poland [24,25]. 

Numerous public campaigns on healthy eating have been carried out by local governments and governmental institutions [26,27]. National public health institutions have also published food-based dietary guidelines for different age groups that promote healthy eating [28]. However, the impact of the educational campaign on public awareness of diet-related diseases and dietary risk factors among adults in Poland is unknown.

This study aimed to assess the level of knowledge on diet-related diseases and dietary risk factors among adults in Poland as well as to identify factors associated with awareness of diet-related diseases and dietary risk factors.

## 2. Materials and Methods

### 2.1. Study Design and Population

Data were obtained from a nationally representative cross-sectional survey carried out by a specialized survey company (Nationwide Research Panel Ariadna) [29] on behalf of the research team. Data were collected between 1 and 4 July 2022 using the computer-assisted web interview (CAWI) method.

A non-probability quota sampling was used [29]. Participants were selected from more than 100,000 registered and verified individual users of the Nationwide Research Panel Ariadna [29]. The stratification model was based on demographic data from the Central Statistical Office of the Republic of Poland and included the following variables: age, gender, and place of residence. A detailed description of the data collection process is presented on the survey company’s website [29].

### 2.2. Measures

The study questionnaire included 20 closed questions on dietary patterns, diet-related diseases, nutrition, health status, and lifestyle. Moreover, questions on sociodemographic characteristics were addressed. During the preparation of the questionnaire, both national and global studies on nutrition and health were analyzed [30,31,32]. 

*Awareness of diet-related diseases*: Respondents were asked about their awareness of diet-related diseases using the following question: “What do you think are diet-related diseases: (1) overweight or obesity; (2) diabetes mellitus; (3) arterial hypertension; (4) myocardial infarction; (5) stroke; (6) cancer (e.g., colorectal or pancreatic cancer); (7) osteoporosis; (8) tooth decay?” with two possible answers: “Yes” or “No”. In this study, overweight/obesity was considered a disease rather than a risk factor because this condition is listed in the International Classification of Diseases (ICD-10) code E66—Overweight and obesity overweight [33]. 

*Awareness of dietary risk factors*: Respondents were asked about their awareness of dietary risk factors using the question: “Which of the following dietary patterns can lead to the development of the diseases: (1) excessive caloric intake (caloric intake > energy expenditure); (2) excessive consumption of sugar and salt; (3) excessive consumption of saturated fatty acids and trans isomers; (4) too little dietary fiber intake; (5) too little vitamin intake; (6) too little consumption of vegetables and fruits; (7) too little intake of calcium and magnesium; (8) too little consumption of fish and oils?” with two possible answers: “Yes” or “No”.

### 2.3. Statistical Analysis

The data were analyzed with SPSS v.28 (IBM, Armonk, NY, USA). The distribution of categorical variables was shown by frequencies and proportions. Cross-tabulations and chi-squared tests were used to compare categorical variables. 

Associations between sociodemographic factors and awareness of (1) diet-related diseases and (2) dietary risk factors were analyzed using multivariable logistic regression models. In simple logistic regression analyses, all variables were considered separately. Multivariable logistic regression analyses included all the variables significantly associated with awareness of diet-related diseases and dietary risk factors in particular models.

The strength of association was measured by the odds ratio (OR) and 95% confidence intervals (95%CI). Statistical inference was based on the criterion *p* < 0.05.

### 2.4. Ethics

Participation in the study was voluntary and anonymous. Informed consent was collected from all the participants. The study was conducted according to the guidelines of the Declaration of Helsinki and approved by the Ethical Review Board at the Medical University of Warsaw, Poland (approval number AKBE/176/2022; date of approval: 13 June 2022).

## 3. Results

### 3.1. Characteristics of the Study Population

Data were received from 1070 individuals (53.3% females) aged 18–89 years. More than half of respondents were married (50.5%), 43.4% had higher education, and one-third lived in rural areas. Characteristics of the study population are presented in Table 1.

### 3.2. Public Awareness of Diet-Related Diseases and Dietary Risk Factors

Out of eight diet-related diseases included in this study, overweight/obesity was the most recognized (85.0%). Three-quarters of respondents were aware that unhealthy diet causes diabetes mellitus. Moreover, a substantial percentage of respondents were aware that diet is an important risk factor in cardiovascular diseases such as arterial hypertension (68.2%) and myocardial infarction (59.1%) Moreover, more than half of respondents indicated cancer (55.9%) as a diet-related disease. Stroke (26.2%) and osteoporosis (17.9%) were the least recognized diet-related diseases (Table 2).

Out of eight dietary risk factors included in this study, excessive consumption of sugar and salt (73.4%) was the most recognized dietary risk factor. Almost two-thirds of respondents indicated too little consumption of vegetables and fruits as a dietary risk factor (62.7%). Less than half of respondents were aware that (1) too little vitamin intake, (2) too little intake of calcium and magnesium, (3) too little consumption of fish and oils, and (4) too little dietary fiber intake can lead to the development of diseases (Table 2).

Respondents with higher education and those with chronic diseases had the highest knowledge of diet-related diseases (Table 3). Females compared to males more often declared that unhealthy diet causes overweight/obesity (90.2% vs. 79.2%, *p* < 0.001), diabetes mellitus (77.5% vs. 70.0%, *p* = 0.01), or tooth decay (58.4% vs. 48.0%, *p* < 0.001). Moreover, the percentage of respondents who indicated that overweight/obesity, arterial hypertension, stroke, cancer, and tooth decay are diet-related diseases differed by age (Table 3). Currently employed/self-employed respondents (active occupational status) more often declared that an unhealthy diet causes myocardial infarction, osteoporosis, and tooth decay (Table 3). 

Respondents with higher education compared to those with lower educational levels had the highest knowledge of all eight dietary risk factors included in this study (Table 4). Moreover, respondents with chronic diseases compared to healthy individuals more often indicated that excessive (1) caloric, (2) sugar and salt, (3) fatty acid and trans isomer intake; too little consumption of vegetables and fruits; or limited consumption of fish and oils are dietary risk factors (Table 4). Females compared to males more often indicated (1) excessive (1) caloric, (2) sugar and salt, (3) fatty acid and trans isomer intake or too little (1) dietary fiber, (2) vegetable and fruit, or (3) fish and oil intake as dietary risk factors. There were no differences in the public awareness of dietary risk factors by occupational status and self-reported financial status (Table 4). Details are presented in Table 4.

### 3.3. Factors Associated with Awareness of Diet-Related Diseases and Dietary Risk Factors

The results of the multivariable logistic regression analyses are presented in Table 5 and Table 6.

A higher educational level was significantly associated (<0.001) with a higher awareness of diet-related diseases (Table 5). Respondents with chronic diseases were more likely to correctly identify diet-related diseases (*p* < 0.05). Females compared to males were more likely to declare that unhealthy diet causes overweight/obesity (OR: 2.22, 95%CI: 1.54–3.18, *p* < 0.001), diabetes mellitus (OR: 1.46, 95%CI: 1.10–1.93, *p* = 0.008), or tooth decay (OR: 1.60, 95%CI: 1.23–2.07, *p* < 0.001). Respondents aged 50–64 were more likely to indicate overweight/obesity (OR: 2.11, 95%CI:1.18–3.77, *p* = 0.01), arterial hypertension (OR: 1.74, 95%CI: 1.17–2.61, *p* = 0.01), stroke (OR: 1.54, 95%CI: 1.04–2.28, *p* = 0.03), and cancer (OR: 1.70, 95%CI: 1.24–2.35, *p* = 0.01) as diet-related diseases. Respondents below 50 years of age were more likely to indicate tooth decay as a diet-related disease (*p* < 0.05). Respondents who had never been married (OR: 1.60, 95%CI: 1.18–2.17, *p* = 0.002), as well as those who lived with at least one person (OR: 1.57, 95%CI: 1.07–2.30, *p* = 0.02), were more likely to declare that unhealthy diet causes tooth decay. Respondents who lived in cities from 20,000 to 99,999 residents were more likely to indicate osteoporosis as a diet-related disease (OR: 1.95, 95%CI: 1.12–3.39, *p* = 0.02). Occupationally active individuals were more likely to declare that an unhealthy diet causes myocardial infarction (OR: 1.58, 95%CI: 1.21–2.07, *p* < 0.001). Moreover, those with moderate finances were more aware of the link between diet and cancer (OR: 1.56, 95%CI: 1.11–2.19, *p* = 0.01) compared to those with bad financial status. Details are presented in Table 5.

A higher educational level was significantly associated (<0.001) with a higher awareness of dietary risk factors (Table 6). Respondents with chronic diseases were more aware of six out of eight analyzed dietary risk factors (Table 6). Females compared to males were more likely to declare that excessive caloric intake (OR: 1.57, 95%CI: 1.22–2.01, *p* < 0.001), excessive consumption of sugar and salt (OR: 1.55, 95%CI: 1.17–2.05, *p* = 0.002), too little dietary fiber intake (OR: 1.38, 95%CI: 1.07–1.78, *p* = 0.01), too little consumption of vegetables and fruits (OR: 1.38, 95%CI: 1.05–1.79, *p* = 0.02), or too little consumption of fish and oils (OR: 1.36, 95%CI: 1.06–1.74, *p* = 0.02) increases risk for diet-related diseases. Respondents aged 65 and over were more likely to indicate that low consumption of vegetables and fruits is a dietary risk factor (OR: 1.68, 95%CI: 1.05–2.69, *p* = 0.03). Those aged 35–49 years were more likely to indicate that too little consumption of fish and oils (OR: 1.41, 95%CI: 1.02–1.95, *p* = 0.04) increased the risk for diet-related diseases. Respondents who lived in the largest cities (above 500,000 residents) were more likely to indicate that too little vitamin intake causes diseases (OR: 1.63, 95%CI: 1.09–2.44, *p* = 0.02). Those with good or moderate financial status were more likely to indicate that too little consumption of vegetables and fruits increases the risk for diseases compared to those with a bad financial situation (*p* < 0.05). There was no influence of marital status, having children, the number of household members, or occupational status on public awareness of dietary risk factors (Table 6).

## 4. Discussion

This is the first study on public awareness of diet-related diseases and dietary risk factors that was carried out on a representative sample of adults in Poland. Findings from this study revealed significant gaps in public awareness of diet-related diseases and dietary risk factors. Most respondents were aware that an unhealthy diet contributes to overweight/obesity and cardiovascular diseases, and a substantial percentage of respondents were not aware that an unhealthy diet increases risk for cancer and osteoporosis. Moreover, less than half of respondents correctly indicated that too little calcium, magnesium, fish, oil, dietary fiber, or vitamin intake are dietary risk factors. Out of 11 factors analyzed in this study, higher education and the presence of chronic diseases were the most important factors associated with a higher level of awareness of diet-related diseases and dietary risk factors.

An unhealthy diet is a modifiable risk factor for numerous NCDs, including cardiometabolic disorders [1,2,3,4,5]. The pathogenesis of diet-related diseases is complex and depends on dietary risk factors [6,7]. Out of eight diet-related diseases analyzed in this study, overweight and obesity was the most recognized group of diseases. The link between diet and weight gain is a well-known fact, so the high percentage of respondents who were aware that overweight and obesity are diet-related diseases may result from general knowledge of biology and nutrition. Findings from this showed that one-quarter of respondents were not aware that an unhealthy diet may increase the risk for diabetes mellitus. The global burden of diabetes is increasing, mostly due to lifestyle changes and the epidemic of obesity [34]. The global nutrition transition also contributes to the epidemic of diabetes, especially in low- and middle-income countries [12,13]. Due to the high social and economic burden of diabetes, further activities are needed to increase public knowledge on diet and its role in the development of type 2 diabetes [1,6]. Cardiovascular diseases are the leading cause of death globally [35]. Regular consumption of fruits and vegetables, whole grains, fish, and low fat significantly reduces the risk of cardiovascular diseases [36,37]. In this study, most of the respondents were aware that arterial hypertension and myocardial infarction are diabetes-related diseases, but only one-quarter of respondents were aware that an unhealthy diet increases the risk of stroke. Numerous studies showed that a diet high in cholesterol, saturated fats, and trans fats increases the risk of stroke [35,36,37]. A relatively high percentage of respondents (53.6%) was aware that an unhealthy diet may lead to tooth decay. In recent years there have been numerous public campaigns on sugar intake and oral health [38], especially those targeted at children and their parents, which may lead to an increase in public knowledge on tooth decay and the reasons behind it. Findings from this study also showed that almost half of adults in Poland were not aware of the link between diet and cancer. Specific dietary components or nutrients (e.g., high salt intake, highly processed foods, and high-calorie foods) are associated with increases in cancer risk (especially colorectal cancer, stomach cancer, breast cancer, and lung cancer) [39,40]. It is estimated that diet represents up to 35% of risk factors that contribute to the onset of cancer [40]. Public health interventions are needed to increase public awareness of dietary risk factors for cancer, both in the general population as well as among cancer survivors. A diet rich in calcium, vitamin D, and protein can help reduce the risk of osteoporosis [41]. In this study, less than one-fifth of respondents were aware that osteoporosis is a diet-related disease. Osteoporosis is becoming increasingly prevalent with the aging of the population, so further educational activities are needed to increase public awareness of risk factors for osteoporosis, especially among females aged 50 and over [42]. 

In this study, excessive consumption of sugar and salt was the most recognized dietary risk factor. In 2013, the World Health Organization encouraged the Member States to implement national policies on salt reduction (by 30% by 2025) [43]. Moreover, different financial, information, defaults, and availability of sugar-sweetened beverage reduction policies were adopted across the world [44]. In 2021, Poland implemented a sugar tax and started a nationwide educational campaign on the health consequences of sugar intake. We can hypothesize that the implementation of the sugar tax had an impact on public awareness of dietary risk factors. Numerous dietary guidelines underline the importance of vegetables and fruit intake [45]. Despite the widespread education on the role of vegetables and fruits in diet, still more than one-third of adults in Poland were not aware of the link between low fruit and vegetable consumption and risk for diseases. Findings from this study revealed a substantial gap in public awareness of the importance of dietary fiber intake, calcium, and magnesium intake, as well as consumption of fish and oil. Policymakers should implement policies that promote the consumption of products rich in dietary fiber as well as fish and oil. Financial barriers should be removed to provide easy access to these food groups. 

Previously published data showed that elderly people with a higher educational level, who lived in urban areas, and who had higher financial status have better dietary knowledge [46,47,48]. In this study, a higher educational level was associated with a higher level of awareness of diet-related diseases and dietary risk factors, which is in line with the previously published data. Moreover, in this study individuals with chronic diseases had a higher level of awareness of diet-related diseases and dietary risk factors. Healthy dietary patterns play an important role in chronic disease prevention and management [49]. We can hypothesize that individuals with chronic diseases were informed about a healthy diet and its role in disease management, so this group has a higher level of dietary knowledge. In this study, females were more likely to correctly indicate diet-related diseases and dietary risk factors. This finding is in line with the gender differences between males and females concerning dietary intake and eating behaviors [50,51]. In this study, there was no influence of marital status, having children, the number of household members, or occupational status on public awareness of dietary risk factors, which may result from the generally low level of knowledge on dietary risk factors among adults in Poland. Moreover, sociodemographic differences in public awareness of diet-related diseases and dietary risk factors point to inequalities and barriers to accessing the knowledge that should be removed by public health authorities and policymakers.

This study has several practical implications. First, comprehensive characteristics of public awareness of diet-related diseases and dietary risk factors presented in this study may be used by healthcare professionals to plan and develop public campaigns on healthy eating. Educational campaigns on dietary risk factors for cancer should be considered a priority action. Second, sociodemographic differences in the level of knowledge on diet-related diseases and dietary risk factors presented in this study underline an urgent need for public health actions aimed at limiting inequalities in nutritional knowledge by gender, age, education, and socioeconomic status. The use of new technologies such as mobile applications and wearables should be considered as a tool supporting nutritional education [52]. Third, despite the significant socio-economic development of Poland during the past three decades, substantial gaps in public awareness of dietary risk factors were observed. Long-term research is needed to regularly monitor eating habits and dietary patterns among citizens of Poland. Findings from this study may be used by other CEE countries to compare nutritional knowledge in different populations with similar historical and socioeconomic backgrounds.

This study has several limitations. First, the list of diet-related diseases and dietary risk factors was limited to the eight most common types, based on the literature review (including the National Institute of Public Health—National Institute of Hygiene database and Institute for Health Metrics and Evaluation datasets) [24,25]. Second, dietary habits and consumption of major food groups were not assessed. Moreover, data on weight and high were not collected, so the calculation of body mass index was missed. As this study was carried out using computer-assisted web interviews, the abovementioned data were not collected due to the high risk of bias. The CAWI method excludes the possibility of interaction with the respondent and is limited to Internet users, but more than 90% of households in Poland have Internet access [53]. Nevertheless, despite these limitations, this is the first study on public awareness of diet-related diseases and dietary risk factors that was carried out among adults in Poland.

## 5. Conclusions

This study demonstrated low public awareness of diet-related diseases and dietary risk factors among adults in Poland. A substantial gap in public awareness of diet-related diseases and dietary risk factors by socioeconomic factors was observed. Educational level and presence of chronic diseases were the most important factors associated with public awareness of diet-related diseases and dietary risk factors. Regular monitoring of public awareness of diet-related diseases and dietary risk factors is necessary to improve the effectiveness of educational campaigns on eating habits.

## Figures and Tables

**Table 1:** Characteristics of the study population (*n* = 1070).

Variable	*n*	%
Gender		
Female	570	53.3
Male	500	46.7
Age (years)		
18–34	345	32.2
35–49	287	26.8
50–64	282	26.4
65+	156	14.6
Educational level		
Primary	24	2.2
Vocational	107	10.0
Secondary	475	44.4
Higher	464	43.4
Marital status		
Single	229	21.4
Married	540	50.5
Informal relationship	174	16.3
Divorced	43	4.0
Widowed	84	7.9
Having children		
Yes	677	63.3
No	393	36.7
Number of household members		
Living alone	147	13.7
2 or more	923	86.3
Place of residence		
Rural	357	33.4
City below 20,000 residents	135	12.6
City from 20,000 to 99,999 residents	227	21.2
City from 100,000 to 499,999 residents	202	18.9
City above 500,000 residents	149	13.9
Occupational status		
Active	666	62.2
Passive	404	37.8
Self-reported economic status		
Rather good, good, or very good	410	38.3
Moderate/difficult to tell	430	40.2
Rather bad, bad, or very good	230	21.5
Presence of chronic diseases		
Yes	481	45.0
No	589	55.0
Self-reported health status		
Rather good, good, or very good	472	44.1
Moderate/difficult to tell	502	46.9
Rather bad, bad, or very good	96	9.0

**Table 2:** Respondents’ knowledge regarding diet-related diseases and dietary risk factors (*n* = 1070).

	Overall (*n* = 1070)	
Variable	*n*	%
**What do you think are diet-related diseases? (multiple-choice format; positive answers)**		
Overweight or obesity	910	85.0
Diabetes mellitus	792	74.0
Arterial hypertension	730	68.2
Myocardial infarction	632	59.1
Stroke	280	26.2
Cancer	598	55.9
Osteoporosis	191	17.9
Tooth decay	573	53.6
**Which of the following dietary patterns can lead to the development of diseases?** **(multiple-choice format; positive answers)**		
Excessive caloric intake	538	50.3
Excessive consumption of sugar and salt	785	73.4
Excessive consumption of saturated fatty acids and trans isomers	575	53.7
Too little dietary fiber intake	413	38.6
Too little vitamin intake	496	46.4
Too little consumption of vegetables and fruits	671	62.7
Too little intake of calcium and magnesium	434	40.6
Too little consumption of fish and oils	467	43.6

**Table 3:** Awareness of diet-related diseases by sociodemographic factors (*n* = 1070).

	**Diet-Related Diseases—Percentage of Respondents Who Answered “Yes”** **by Sociodemographic Factors**							
**Variable**	**Overweight or Obesity**		**Diabetes Mellitus**		**Arterial Hypertension**		**Myocardial Infarction**	
	***n* (%)**	***p***	***n* (%)**	***p***	***n* (%)**	***p***	***n* (%)**	***p***
Gender								
Female	514 (90.2)	**<0.001**	442 (77.5)	**0.01**	402 (70.5)	0.08	350 (61.4)	0.1
Male	396 (79.2)		350 (70.0)		328 (65.6)		282 (56.4)	
Age (years)								
18–34	275 (79.7)	**<0.001**	258 (74.8)	0.7	209 (60.6)	**<0.001**	194 (56.2)	0.2
35–49	237 (82.6)		209 (72.8)		201 (70.0)		181 (63.1)	
50–64	259 (91.8)		214 (75.9)		213 (75.5)		171 (60.6)	
65+	139 (89.1)		111 (71.2)		107 (68.6)		86 (55.1)	
Educational level								
Primary	18 (75.0)	**<0.001**	14 (58.3)	**<0.001**	11 (45.8)	**<0.001**	12 (50.0)	**0.003**
Vocational	78 (72.9)		56 (52.3)		61 (57.0)		51 (47.7)	
Secondary	402 (84.6)		355 (74.7)		307 (64.6)		269 (56.6)	
Higher	412 (88.8)		367 (79.1)		351 (75.6)		300 (64.7)	
Marital status								
Single	183 (79.9)	0.1	176 (76.9)	0.3	144 (62.9)	0.4	128 (55.9)	0.8
Married	469 (86.9)		393 (72.8)		380 (70.4)		324 (60.0)	
Informal relationship	146 (83.9)		134 (77.0)		120 (69.0)		106 (60.9)	
Divorced	39 (90.7)		33 (76.7)		29 (67.4)		25 (58.1)	
Widowed	73 (86.9)		56 (66.7)		57 (67.9)		49 (58.3)	
Having children								
Yes	596 (88.0)	**<0.001**	496 (73.3)	0.5	478 (70.6)	**0.03**	401 (59.2)	0.9
No	314 (79.9)		296 (75.3)		252 (64.1)		231 (58.8)	
Number of household members								
Living alone	122 (83.0)	0.5	107 (72.8)	0.7	99 (67.3)	0.8	80 (54.4)	0.2
2 or more	788 (85.4)		685 (74.2)		631 (68.4)		552 (59.8)	
Place of residence								
Rural	299 (83.8)	0.5	262 (73.4)	**0.04**	229 (64.1)	0.1	203 (56.9)	0.2
City below 20,000 residents	110 (81.5)		91 (67.4)		89 (65.9)		83 (61.5)	
City from 20,000 to 99,999 residents	194 (85.5)		170 (74.9)		158 (69.6)		140 (61.7)	
City from 100,000 to 499,999 residents	178 (88.1)		145 (71.8)		141 (69.8)		109 (54.0)	
City above 500,000 residents	129 (86.6)		124 (83.2)		113 (75.8)		97 (65.1)	
Occupational status								
Active	560 (84.1)	0.3	488 (73.3)	0.5	467 (70.1)	0.09	416 (62.5)	**0.004**
Passive	350 (86.6)		304 (75.2)		263 (65.1)		216 (53.5)	
Self-reported financial status								
Rather good, good, or very good	352 (85.9)	0.7	311 (75.9)	0.3	282 (68.8)	0.4	241 (58.8)	0.9
Moderate/difficult to tell	366 (85.1)		320 (74.4)		284 (66.0)		254 (59.1)	
Rather bad, bad, or very good	192 (83.5)		161 (70.0)		164 (71.3)		137 (59.6)	
Presence of chronic diseases								
Yes	434 (90.2)	**<0.001**	378 (78.6)	**0.002**	357 (74.2)	**<0.001**	310 (64.4)	**0.001**
No	476 (80.8)		414 (70.3)		373 (63.3)		322 (54.7)	
Self-reported health status								
Rather good, good, or very good	406 (86.0)	0.7	351 (74.4)	0.9	319 (67.6)	0.9	270 (57.2)	0.5
Moderate/difficult to tell	424 (84.5)		369 (73.5)		346 (68.9)		302 (60.2)	
Rather bad, bad, or very good	80 (83.3)		72 (75.0)		65 (67.7)		60 (62.5)	
**Variable**	**Stroke**		**Cancer**		**Osteoporosis**		**Tooth Decay**	
	***n* (%)**	***p***	***n* (%)**	***p***	***n* (%)**	***p***	***n* (%)**	***p***
Gender								
Female	157 (27.5)	0.3	334 (58.6)	0.057	106 (18.6)	0.5	333 (58.4)	**<0.001**
Male	123 (24.6)		264 (52.8)		85 (17.0)		240 (48.0)	
Age (years)								
18–34	67 (19.4)	**0.01**	169 (49.0)	**0.01**	61 (17.7)	0.9	211 (61.2)	**<0.001**
35–49	88 (30.7)		166 (57.8)		55 (19.2)		160 (55.7)	
50–64	80 (28.4)		175 (62.1)		48 (17.0)		140 (49.6)	
65+	45 (28.8)		88 (56.4)		27 (17.3)		62 (39.7)	
Educational level								
Primary	3 (12.5)	**<0.001**	9 (37.5)	**<0.001**	2 (8.3)	**<0.001**	13 (54.2)	**<0.001**
Vocational	13 (12.1)		40 (37.4)		13 (12.1)		33 (30.8)	
Secondary	109 (22.9)		251 (52.8)		67 (14.1)		238 (50.1)	
Higher	155 (33.4)		298 (64.2)		109 (23.5)		289 (62.3)	
Marital status								
Single	53 (23.1)	0.2	119 (52.0)	0.4	41 (17.9)	0.1	130 (56.8)	**<0.001**
Married	140 (25.9)		314 (58.1)		88 (16.3)		271 (50.2)	
Informal relationship	57 (32.8)		96 (55.2)		42 (24.1)		116 (66.7)	
Divorced	10 (23.3)		20 (46.5)		4 (9.3)		16 (37.2)	
Widowed	20 (23.8)		49 (58.3)		16 (19.0)		40 (47.6)	
Having children								
Yes	180 (26.6)	0.7	376 (55.5)	0.8	109 (16.1)	0.05	350 (51.7)	0.1
No	100 (25.4)		222 (56.5)		82 (20.9)		223 (56.7)	
Number of household members								
Living alone	38 (25.9)	0.9	76 (51.7)	0.3	38 (25.9)	**0.006**	67 (45.6)	**0.04**
2 or more	242 (26.2)		522 (56.6)		153 (16.6)		506 (54.8)	
Place of residence								
Rural	84 (23.5)	0.2	193 (54.1)	0.7	62 (17.4)	0.1	193 (54.1)	0.2
City below 20,000 residents	38 (28.1)		77 (57.0)		18 (13.3)		60 (44.4)	
City from 20,000 to 99,999 residents	63 (27.8)		129 (56.8)		53 (23.3)		124 (54.6)	
City from 100,000 to 499,999 residents	47 (23.3)		120 (59.4)		36 (17.8)		108 (53.5)	
City above 500,000 residents	48 (32.2)		79 (53.0)		22 (14.8)		88 (59.1)	
Occupational status								
Active	186 (27.9)	0.09	377 (56.6)	0.5	131 (19.7)	**0.046**	382 (57.4)	**0.001**
Passive	94 (23.3)		221 (54.7)		60 (14.9)		191 (47.3)	
Self-reported financial status								
Rather good, good, or very good	114 (27.8)	0.5	228 (55.6)	**0.01**	70 (17.1)	0.3	231 (56.3)	0.3
Moderate/difficult to tell	104 (24.2)		260 (60.5)		72 (16.7)		227 (52.8)	
Rather bad, bad, or very good	62 (27.0)		110 (47.8)		49 (21.3)		115 (50.0)	
Presence of chronic diseases								
Yes	145 (30.1)	**0.01**	298 (62.0)	**<0.001**	94 (19.5)	0.2	276 (57.4)	**0.02**
No	135 (22.9)		300 (50.9)		97 (16.5)		297 (50.4)	
Self-reported health status								
Rather good, good, or very good	119 (25.2)	0.8	265 (56.1)	0.06	86 (18.2)	0.8	267 (56.6)	0.2
Moderate/difficult to tell	136 (27.1)		290 (57.8)		90 (17.9)		256 (51.0)	
Rather bad, bad, or very good	25 (26.0)		43 (44.8)		15 (15.6)		50 (52.1)	

**Table 4:** Awareness of dietary patterns that increase the risk for dietary-related diseases (*n* = 1070).

	**Risk Factors for Diet-Related Diseases—Percentage of Respondents Who Answered “Yes” by Sociodemographic Factors**							
**Variable**	**Excessive Caloric Intake**		**Excessive Consumption of Sugar and Salt**		**Excessive Consumption of Saturated Fatty Acids and Trans Isomers**		**Too Little Dietary** **Fiber Intake**	
	***n* (%)**	***p***	***n* (%)**	***p***	***n* (%)**	***p***	***n* (%)**	***p***
Gender								
Female	317 (55.6)	**<0.001**	443 (77.7)	**<0.001**	326 (57.2)	**0.02**	240 (42.1)	**0.01**
Male	221 (44.2)		342 (68.4)		249 (49.8)		173 (34.6)	
Age (years)								
18–34	169 (49.0)	0.6	244 (70.7)	0.1	165 (47.8)	**0.048**	121 (35.1)	0.2
35–49	139 (48.4)		202 (70.4)		158 (55.1)		109 (38.0)	
50–64	151 (53.5)		218 (77.3)		165 (58.5)		112 (39.7)	
65+	79 (50.6)		121 (77.6)		87 (55.8)		71 (45.5)	
Educational level								
Primary	10 (41.7)	**<0.001**	14 (58.3)	**<0.001**	6 (25.0)	**<0.001**	7 (29.2)	**<0.001**
Vocational	32 (29.9)		64 (59.8)		43 (40.2)		29 (27.1)	
Secondary	221 (46.5)		341 (71.8)		240 (50.5)		163 (34.3)	
Higher	275 (59.3)		366 (78.9)		286 (61.6)		214 (46.1)	
Marital status								
Single	115 (50.2)	0.9	155 (67.7)	0.2	116 (50.7)	0.6	84 (36.7)	0.1
Married	271 (50.2)		401 (74.3)		288 (53.3)		198 (36.7)	
Informal relationship	90 (51.7)		132 (75.9)		100 (57.5)		79 (45.4)	
Divorced	19 (44.2)		31 (72.1)		26 (60.5)		14 (32.6)	
Widowed	43 (51.2)		66 (78.6)		45 (53.6)		38 (45.2)	
Having children								
Yes	344 (50.8)	0.6	511 (75.5)	**0.04**	369 (54.5)	0.5	271 (40.0)	0.2
No	194 (49.4)		274 (69.7)		206 (52.4)		142 (36.1)	
Number of household members								
Living alone	76 (51.7)	0.7	102 (69.4)	0.2	86 (58.5)	0.2	54 (36.7)	0.6
2 or more	462 (50.1)		683 (74.0)		489 (53.0)		359 (38.9)	
Place of residence								
Rural	169 (47.3)	**0.03**	249 (69.7)	0.2	183 (51.3)	0.2	131 (36.7)	0.2
City below 20,000 residents	60 (44.4)		100 (74.1)		64 (47.4)		43 (31.9)	
City from 20,000 to 99,999 residents	110 (48.5)		167 (73.6)		127 (55.9)		96 (42.3)	
City from 100,000 to 499,999 residents	109 (54.0)		150 (74.3)		118 (58.4)		77 (38.1)	
City above 500,000 residents	90 (60.4)		119 (79.9)		83 (55.7)		66 (44.3)	
Occupational status								
Active	336 (50.5)	0.9	493 (74.0)	0.5	366 (55.0)	0.3	259 (38.9)	0.8
Passive	202 (50.0)		292 (72.3)		209 (51.7)		154 (38.1)	
Self-reported financial status								
Rather good, good, or very good	215 (52.4)	0.5	302 (73.7)	0.9	224 (54.6)	0.4	163 (39.8)	0.7
Moderate/difficult to tell	213 (49.5)		315 (73.3)		236 (54.9)		167 (38.8)	
Rather bad, bad, or very good	110 (47.8)		168 (73.0)		115 (50.0)		83 (36.1)	
Presence of chronic diseases								
Yes	279 (58.0)	**<0.001**	383 (79.6)	**<0.001**	283 (58.8)	**0.003**	208 (43.2)	**0.01**
No	259 (44.0)		402 (68.3)		292 (49.6)		205 (34.8)	
Self-reported health status								
Rather good, good, or very good	241 (51.1)	0.9	350 (74.2)	0.5	251 (53.2)	0.08	189 (40.0)	0.7
Moderate/difficult to tell	248 (49.4)		361 (71.9)		282 (56.2)		187 (37.3)	
Rather bad, bad, or very good	49 (51.0)		74 (77.1)		42 (43.8)		37 (38.5)	
**Variable**	**Too Little Vitamin Intake**		**Too Little Consumption of Vegetables and Fruits**		**Too Little Intake of Calcium and Magnesium**		**Too Little Consumption of Fish and Oils**	
	***n* (%)**	***p***	***n* (%)**	***p***	***n* (%)**	***p***	***n* (%)**	***p***
Gender								
Female	279 (48.9)	0.07	378 (66.3)	**0.01**	240 (42.1)	0.3	269 (47.2)	**0.01**
Male	217 (43.4)		293 (58.6)		194 (38.8)		198 (39.6)	
Age (years)								
18–34	183 (53.0)	**0.004**	190 (55.1)	**<0.001**	145 (42.0)	0.9	127 (36.8)	**0.01**
35–49	137 (47.7)		178 (62.0)		113 (39.4)		131 (45.6)	
50–64	110 (39.0)		191 (67.7)		111 (39.4)		130 (46.1)	
65+	66 (42.3)		112 (71.8)		65 (41.7)		79 (50.6)	
Educational level								
Primary	9 (37.5)	**<0.001**	11 (45.8)	**<0.001**	8 (33.3)	**0.03**	6 (25.0)	**0.003**
Vocational	34 (31.8)		52 (48.6)		35 (32.7)		35 (32.7)	
Secondary	205 (43.2)		295 (62.1)		180 (37.9)		200 (42.1)	
Higher	248 (53.4)		313 (67.5)		211 (45.5)		226 (48.7)	
Marital status								
Single	103 (45.0)	**<0.001**	137 (59.8)	0.5	83 (36.2)	**0.02**	85 (37.1)	0.2
Married	225 (41.7)		340 (63.0)		219 (40.6)		238 (44.1)	
Informal relationship	107 (61.5)		107 (61.5)		86 (49.4)		86 (49.4)	
Divorced	16 (37.2)		31 (72.1)		11 (25.6)		20 (46.5)	
Widowed	45 (53.6)		56 (66.7)		35 (41.7)		38 (45.2)	
Having children								
Yes	303 (44.8)	0.2	445 (65.7)	**0.007**	271 (40.0)	0.6	310 (45.8)	0.06
No	193 (49.1)		226 (57.5)		163 (41.5)		157 (39.9)	
Number of household members								
Living alone	60 (40.8)	0.1	95 (64.6)	0.6	51 (34.7)	0.1	62 (42.2)	0.7
2 or more	436 (47.2)		576 (62.4)		383 (41.5)		405 (43.9)	
Place of residence								
Rural	149 (41.7)	**0.02**	205 (57.4)	0.08	138 (38.7)	0.2	141 (39.5)	0.06
City below 20,000 residents	54 (40.0)		81 (60.0)		45 (33.3)		50 (37.0)	
City from 20,000 to 99,999 residents	113 (49.8)		152 (67.0)		99 (43.6)		108 (47.6)	
City from 100,000 to 499,999 residents	98 (48.5)		135 (66.8)		85 (42.1)		98 (48.5)	
City above 500,000 residents	82 (55.0)		98 (65.8)		67 (45.0)		70 (47.0)	
Occupational status								
Active	324 (48.6)	0.053	406 (61.0)	0.1	285 (42.8)	0.056	285 (42.8)	0.5
Passive	172 (42.6)		265 (65.6)		149 (36.9)		182 (45.0)	
Self-reported financial status								
Rather good, good, or very good	192 (46.8)	0.4	263 (64.1)	0.06	174 (42.4)	0.4	170 (41.5)	0.5
Moderate/difficult to tell	206 (47.9)		279 (64.9)		175 (40.7)		192 (44.7)	
Rather bad, bad, or very good	98 (42.6)		129 (56.1)		85 (37.0)		105 (45.7)	
Presence of chronic diseases								
Yes	227 (47.2)	0.6	334 (69.4)	**<0.001**	208 (43.2)	0.1	240 (49.9)	**<0.001**
No	269 (45.7)		337 (57.2)		226 (38.4)		227 (38.5)	
Self-reported health status								
Rather good, good, or very good	240 (50.8)	**0.02**	314 (66.5)	0.05	194 (41.1)	0.6	212 (44.9)	0.5
Moderate/difficult to tell	219 (43.6)		296 (59.0)		206 (41.0)		218 (43.4)	
Rather bad, bad, or very good	37 (38.5)		61 (63.5)		34 (35.4)		37 (38.5)	

**Table 5:** Factors associated with awareness of diet-related diseases (*n* = 1070).

	**Factors Associated with Awareness of Diet–Related Diseases**											
**Variable**	**Overweight or Obesity**				**Diabetes Mellitus**				**Arterial Hypertension**			
	**Simple Logistic Regression**		**Multivariable Logistic Regression**		**Simple Logistic Regression**		**Multivariable Logistic Regression**		**Simple Logistic Regression**		**Multivariable Logistic Regression**	
	***p***	**OR (95%CI)**	***p***	**OR (95%CI)**	***p***	**OR (95%CI)**	***p***	**OR (95%CI)**	***p***	**OR (95%CI)**	***p***	**OR (95%CI)**
Gender												
Female	**<0.001**	2.41 (1.70–3.42)	**<0.001**	2.22 (1.54–3.18)	**0.005**	1.48 (1.13–1.95)	**0.008**	1.46 (1.10–1.93)	0.08	1.26 (0.97–1.62)		
Male		Reference		Reference		Reference		Reference		Reference		
Age (years)												
18–34		Reference		Reference	0.4	1.20 (0.79-1.84)				Reference		Reference
35–49	0.4	1.21 (0.81–1.80)	0.8	1.50 (0.77–2.94)	0.7	1.09 (0.70–1.68)			**0.01**	1.52 (1.09–2.12)	0.06	1.06 (0.66–1.68)
50–64	**<0.001**	2.87 (1.74–4.73)	**0.01**	2.11 (1.18–3.77)	0.3	1.28 (0.82–1.98)			**<0.001**	2.01 (1.42–2.84)	**0.01**	1.74 (1.17–2.61)
65+	**0.01**	2.08 (1.18–3.67)	0.2	1.50 (0.68–1.69)		Reference			0.09	1.42 (0.95–2.12)	0.8	1.06 (0.66–1.68)
Having higher education												
Yes	**0.003**	1.72 (1.20–2.45)	**0.001**	1.84 (1.28–2.67)	**<0.001**	1.61 (1.21–2.14)	**0.001**	1.60 (1.20–2.14)	**<0.001**	1.86 (1.42–2.43)	**<0.001**	1.89 (1.43–2.50)
No		Reference		Reference		Reference		Reference		Reference		Reference
Ever married												
Yes	**0.02**	1.52 (1.08–2.13)	0.5	0.84 (0.52–1.36)	0.1	0.78 (0.59–1.04)			0.1	1.22 (0.94–1.59)		
No		Reference		Reference		Reference				Reference		
Having children												
Yes	**<0.001**	1.85 (1.32–2.60)	0.2	1.38 (0.85–2.24)	0.4	0.90 (0.68–1.20)			**0.03**	1.34 (1.03–1.75)	0.7	1.06 (0.77–1.45)
No		Reference		Reference		Reference				Reference		Reference
Number of household members												
Living alone	0.5	0.84 (0.52–1.33)			0.7	0.93 (0.63–1.38)			0.8	0.95 (0.66–1.38)		
2 or more		Reference				Reference				Reference		
Place of residence												
Rural		Reference				Reference		Reference		Reference		Reference
City below 20,000 residents	0.5	0.85 (0.51–1.43)			0.2	0.75 (0.49–1.15)	0.2	0.73 (0.47–1.12)	0.7	1.08 (0.71–1.64)	0.8	1.07 (0.70–1.64)
City from 20,000 to 99,999 residents	0.6	1.14 (0.72–1.81)			0.7	1.08 (0.74–1.58)	0.8	0.95 (0.65–1.41)	0.2	1.28 (0.90–1.83)	0.6	1.12 (0.77–1.62)
City from 100,000 to 499,999 residents	0.2	1.44 (0.86–2.40)			0.7	0.92 (0.63–1.36)	0.4	0.85 (0.58–1.25)	0.2	1.29 (0.89–1.87)	0.4	1.16 (0.79–1.70)
City above 500,000 residents	0.4	1.25 (0.72–2.17)			**0.02**	1.80 (1.10–2.94)	0.1	1.53 (0.93–2.51)	**0.01**	1.75 (1.14–2.71)	0.1	1.43 (0.91–2.24)
Occupational status												
Active		Reference				Reference				Reference		
Passive	0.3	1.23 (0.86–1.75)			0.5	1.11 (0.84–1.47)			0.09	0.80 (0.61–1.03)		
Self-reported financial status												
Rather good, good or very good	0.4	1.20 (0.77–1.88)			0.1	1.35 (0.94–1.93)			0.5	0.89 (0.62–1.26)		
Moderate/difficult to tell	0.6	1.13 (0.73–1.75)			0.2	1.25 (0.87–1.78)			0.2	0.78 (0.55–1.11)		
Rather bad, bad or very good		Reference				Reference				Reference		
Presence of chronic diseases												
Yes	**<0.001**	2.20 (1.52–3.16)	**0.005**	1.77 (1.19–2.63)	**0.002**	1.55 (1.17–2.05)	**0.004**	1.52 (1.14–2.02)	**<0.001**	1.67 (1.28–2.17)	**0.002**	1.58 (1.18–2.10)
No		Reference		Reference		Reference		Reference		Reference		Reference
Self-reported health status												
Rather good, good or very good	0.5	1.23 (0.68–2.23)			0.9	0.97 (0.58–1.60)			0.9	0.99 (0.62–1.59)		
Moderate/difficult to tell	0.8	1.09 (0.60–1.96)			0.8	0.93 (0.56–1.53)			0.8	1.06 (0.66–1.69)		
Rather bad, bad or very good		Reference				Reference				Reference		
**Variable**	**Myocardial Infarction**				**Stroke**				**Cancer**			
	**Simple Logistic Regression**		**Multivariable Logistic Regression**		**Simple Logistic Regression**		**Multivariable Logistic Regression**		**Simple Logistic Regression**		**Multivariable Logistic Regression**	
	***p***	**OR (95%CI)**	***p***	**OR (95%CI)**	***p***	**OR (95%CI)**	***p***	**OR (95%CI)**	***p***	**OR (95%CI)**	***p***	**OR (95%CI)**
Gender												
Female	0.1	1.23 (0.96–1.57)			0.3	1.17 (0.89–1.53)			0.057	1.27 (0.99–1.61)		
Male		Reference				Reference				Reference		
Age (years)												
18–34	0.8	1.05 (0.72–1.53)				Reference		Reference		Reference		Reference
35–49	0.1	1.39 (0.94–2.07)			**0.001**	1.84 (1.27–2.65)	**0.002**	1.78 (1.23–2.58)	**0.03**	1.43 (1.04–1.96)	0.09	1.33 (0.96–1.84)
50–64	0.3	1.25 (0.84–1.86)			**0.01**	1.64 (1.13–2.38)	**0.03**	1.54 (1.04–2.28)	**0.001**	1.70 (1.24–2.35)	**0.01**	1.54 (1.10–2.17)
65+		Reference			**0.02**	1.68 (1.09–2.61)	0.2	1.36 (0.86–2.16)	0.1	1.35 (0.92–1.97)	0.7	1.09 (0.72–1.64)
Having higher education												
Yes	**0.001**	1.51 (1.18–1.94)	**0.005**	1.44 (1.12–1.86)	**<0.001**	1.93 (1.47–2.54)	**<0.001**	1.93 (1.45–2.56)	**<0.001**	1.83 (1.43–2.35)	**<0.001**	1.88 (1.46–2.44)
No		Reference		Reference		Reference		Reference		Reference		Reference
Ever married												
Yes	0.6	1.07 (0.83–1.37)			0.5	0.91 (0.69–1.21)			0.2	1.18 (0.92–1.51)		
No		Reference				Reference				Reference		
Having children												
Yes	0.9	1.02 (0.79–1.31)			0.7	1.06 (0.80–1.41)			0.8	0.96 (0.75–1.24)		
No		Reference				Reference				Reference		
Number of household members												
Living alone	0.2	0.80 (0.57–1.14)			0.9	0.98 (0.66–1.46)			0.3	0.82 (0.58–1.17)		
2 or more		Reference				Reference				Reference		
Place of residence												
Rural		Reference				Reference		Reference		Reference		
City below 20,000 residents	0.4	1.21 (0.81–1.82)			0.3	1.27 (0.81–1.99)	0.3	1.26 (0.80–2.00)	0.6	1.13 (0.76–1.68)		
City from 20,000 to 99,999 residents	0.3	1.22 (0.87–1.72)			0.3	1.25 (0.85–1.83)	0.6	1.10 (0.74–1.63)	0.5	1.12 (0.80–1.56)		
City from 100,000 to 499,999 residents	0.5	0.89 (0.63–1.26)			0.9	0.99 (0.66–1.48)	0.6	0.91 (0.60–1.38)	0.2	1.24 (0.88–1.76)		
City above 500,000 residents	0.09	1.42 (0.95–2.10)			**0.04**	1.55 (1.01–2.36)	0.2	1.29 (0.84–2.00)	0.8	0.96 (0.65–1.41)		
Occupational status												
Active	**0.004**	1.45 (1.13–1.86)	**<0.001**	1.58 (1.21–2.07)	0.09	1.28 (0.96–1.70)			0.5	1.08 (0.84–1.39)		
Passive		Reference		Reference		Reference				Reference		
Self-reported financial status												
Rather good, good or very good	0.8	0.97 (0.70–1.35)			0.8	1.04 (0.73–1.50)			0.06	1.37 (0.99–1.89)	0.3	1.22 (0.86–1.75)
Moderate/difficult to tell	0.9	0.98 (0.71–1.36)			0.4	0.86 (0.60–1.25)			**0.002**	1.67 (1.21–2.30)	**0.01**	1.56 (1.11–2.19)
Rather bad, bad or very good		Reference				Reference				Reference		Reference
Presence of chronic diseases												
Yes	**0.001**	1.50 (1.17–1.93)	**<0.001**	1.73 (1.33–2.24)	**0.008**	1.45 (1.10–1.91)	**0.02**	1.42 (1.05–1.92)	**<0.001**	1.57 (1.23–2.00)	**<0.001**	1.80 (1.35–2.40)
No		Reference		Reference		Reference		Reference		Reference		Reference
Self-reported health status												
Rather good, good or very good	0.3	0.80 (0.51–1.26)			0.9	0.96 (0.58–1.58)			**0.04**	1.58 (1.02–2.45)	**0.007**	2.02 (1.21–3.35)
Moderate/difficult to tell	0.7	0.91 (0.58–1.42)			0.8	1.06 (0.64–1.73)			**0.02**	1.69 (1.09–2.62)	**0.01**	1.83 (1.14–2.93)
Rather bad, bad or very good		Reference				Reference				Reference		Reference
**Variable**	**Osteoporosis**						**Tooth Decay**					
	**Simple Logistic Regression**			**Multivariable Logistic Regression**			**Simple Logistic Regression**			**Multivariable Logistic Regression**		
	***p***	**OR (95%CI)**		***p***	**OR (95%CI)**		***p***	**OR (95%CI)**		***p***	**OR (95%CI)**	
Gender												
Female	0.5	1.12 (0.81–1.53)					**<0.001**	1.52 (1.20–1.94)		**<0.001**	1.60 (1.23–2.07)	
Male		Reference						Reference			Reference	
Age (years)												
18–34		Reference					**<0.001**	2.39 (1.62–3.52)		**0.004**	2.07 (1.26–3.41)	
35–49	0.6	1.10 (0.74–1.65)					**0.001**	1.91 (1.29–2.84)		**0.03**	1.73 (1.06–2.83)	
50–64	0.8	0.96 (0.63–1.45)					**0.047**	1.50 (1.01–2.22)		0.2	1.34 (0.86–2.10)	
65+	0.9	0.97 (0.59–1.60)						Reference			Reference	
Having higher education												
Yes	**<0.001**	1.96 (1.43–2.69)		**<0.001**	1.91 (1.38–2.65)		**<0.001**	1.87 (1.46–2.40)		**<0.001**	2.00 (1.54–2.60)	
No		Reference			Reference			Reference			Reference	
Ever married												
Yes	0.07	0.75 (0.54–1.02)						Reference			Reference	
No		Reference					**<0.001**	1.63 (1.27–2.10)		**0.002**	1.60 (1.18–2.17)	
Having children												
Yes	0.05	0.73 (0.53–1.00)					0.1	0.82 (0.64–1.05)				
No		Reference						Reference				
Number of household members												
Living alone	0.007	1.76 (1.17–2.64)						Reference			Reference	
2 or more		Reference					**0.04**	1.45 (1.02–2.06)		**0.02**	1.57 (1.07–2.30)	
Place of residence												
Rural	0.5	1.21 (0.72–2.06)		0.2	1.43 (0.83–2.44)			Reference				
City below 20,000 residents	0.7	0.89 (0.45–1.74)		0.9	1.00 (0.51–1.97)		0.06	0.68 (0.46–1.01)				
City from 20,000 to 99,999 residents	**0.04**	1.76 (1.02–3.04)		**0.02**	1.95 (1.12–3.39)		0.9	1.02 (0.73–1.43)				
City from 100,000 to 499,999 residents	0.4	1.25 (0.70–2.23)		0.3	1.37 (0.76–2.46)		0.9	0.98 (0.69–1.38)				
City above 500,000 residents		Reference			Reference		0.3	1.23 (0.83–1.81)				
Occupational status												
Active	**0.047**	1.40 (1.01–1.96)		0.1	1.30 (0.92–1.83)		**0.001**	1.50 (1.17–1.92)		0.2	1.26 (0.92–1.71)	
Passive		Reference			Reference			Reference			Reference	
Self-reported financial status												
Rather good, good or very good	0.2	0.76 (0.51–1.14)					0.1	1.29 (0.93–1.78)				
Moderate/difficult to tell	0.2	0.74 (0.50-1.11)					0.5	1.12 (0.81-1.54)				
Rather bad, bad or very good		Reference						Reference				
Presence of chronic diseases												
Yes	**0.2**	1.23 (0.90–1.69)					**0.02**	1.32 (1.04–1.69)		**<0.001**	1.85 (1.40–2.44)	
No		Reference						Reference			Reference	
Self-reported health status												
Rather good, good or very good	0.5	1.20 (0.66–2.19)					0.4	1.20 (0.77–1.86)				
Moderate/difficult to tell	0.6	1.18 (0.65–2.14)					0.8	0.96 (0.62–1.48)				
Rather bad, bad or very good		Reference						Reference				

**Table 6:** Awareness of dietary behaviors that increase the risk for diet-related diseases (*n* = 1070).

	**Factors Associated with Awareness of Dietary Behaviors That Increase the Risk for Diet–Related Diseases**											
**Variable**	**Excessive Caloric Intake**				**Excessive Consumption of Sugar and Salt**				**Excessive Consumption of Saturated Fatty Acids and Trans Isomers**			
	**Simple Logistic Regression**		**Multivariable Logistic Regression**		**Simple Logistic Regression**		**Multivariable Logistic Regression**		**Simple Logistic Regression**		**Multivariable Logistic Regression**	
	***p***	**OR (95%CI)**	***p***	**OR (95%CI)**	***p***	**OR (95%CI)**	***p***	**OR (95%CI)**	***p***	**OR (95%CI)**	***p***	**OR (95%CI)**
Gender												
Female	**<0.001**	1.58 (1.24–2.01)	**<0.001**	1.57 (1.22–2.01)	**<0.001**	1.61 (1.23–2.12)	**0.002**	1.55 (1.17–2.05)	**0.02**	1.35 (1.06–1.71)	0.05	1.28 (0.99–1.64)
Male		Reference		Reference		Reference		Reference		Reference		Reference
Age (years)												
18–34	0.7	0.94 (0.64–1.37)		Reference		Reference				Reference		Reference
35–49	0.7	0.92 (0.62–1.35)			0.9	0.98 (0.70–1.39)			0.07	1.34 (0.98–1.83)	0.2	1.27 (0.92–1.75)
50–64	0.6	1.12 (0.76–1.66)			0.06	1.41 (0.98–2.03)			**0.01**	1.54 (1.12–2.11)	0.06	1.38 (0.98–1.93)
65+		Reference			0.1	1.43 (0.92–2.23)			0.1	1.38 (0.94–2.01)	0.4	1.18 (0.79–1.78)
Having higher education												
Yes	**<0.001**	1.90 (1.49–2.43)	**<0.001**	1.94 (1.51–2.50)	**<0.001**	1.67 (1.26–2.21)	**<0.001**	1.67 (1.25–2.23)	**<0.001**	1.76 (1.38–2.25)	**<0.001**	1.81 (1.41–2.33)
No		Reference		Reference		Reference		Reference		Reference		Reference
Ever married												
Yes	0.8	0.96 (0.75–1.23)			0.2	1.19 (0.90–1.57)			0.9	1.01 (0.79–1.29)		
No		Reference				Reference				Reference		
Having children												
Yes	0.6	1.06 (0.83–1.36)			**0.04**	1.34 (1.01–1.76)	0.3	1.15 (0.87–1.54)	0.5	1.09 (0.85–1.40)		
No		Reference				Reference		Reference		Reference		
Number of household members												
Living alone	0.7	1.07 (0.75–1.51)			0.2	0.80 (0.55–1.17)			0.2	1.25 (0.88–1.78)		
2 or more		Reference				Reference				Reference		
Place of residence												
Rural		Reference		Reference		Reference		Reference		Reference		
City below 20,000 residents	0.6	0.89 (0.60–1.33)	0.5	0.86 (0.57–1.29)	0.3	1.24 (0.79–1.94)	0.4	1.21 (0.76–1.90)	0.4	0.86 (0.58–1.27)		
City from 20,000 to 99,999 residents	0.8	1.05 (0.75–1.46)	0.5	0.88 (0.62–1.24)	0.3	1.21 (0.83–1.75)	0.9	1.04 (0.71–1.52)	0.3	1.21 (0.86–1.69)		
City from 100,000 to 499,999 residents	0.1	1.30 (0.92–1.84)	0.3	1.20 (0.84–1.71)	0.3	1.25 (0.85–1.84)	0.5	1.15 (0.77–1.71)	0.1	1.34 (0.94–1.89)		
City above 500,000 residents	**0.008**	1.70 (1.15–2.50)	0.1	1.37 (0.92–2.05)	**0.02**	1.72 (1.09–2.73)	0.1	1.43 (0.89–2.29)	0.4	1.20 (0.81–1.76)		
Occupational status												
Active	0.9	1.02 (0.80–1.30)			0.5	1.09 (0.83–1.44)			0.3	1.14 (0.89–1.46)		
Passive		Reference				Reference				Reference		
Self-reported financial status												
Rather good, good or very good	0.3	1.20 (0.87–1.66)			0.9	1.03 (0.72–1.49)			0.3	1.20 (0.87–1.66)		
Moderate/difficult to tell	0.7	1.07 (0.78–1.48)			0.9	1.01 (0.70–1.45)			0.2	1.22 (0.88–1.68)		
Rather bad, bad or very good		Reference				Reference				Reference		
Presence of chronic diseases												
Yes	**<0.001**	1.76 (1.38–2.25)	**<0.001**	1.79 (1.39–2.31)	**<0.001**	1.82 (1.37–2.41)	**<0.001**	1.77 (1.33–2.37)	**0.003**	1.45 (1.14–1.85)	**0.003**	1.55 (1.16–2.06)
No		Reference		Reference		Reference		Reference		Reference		Reference
Self-reported health status												
Rather good, good or very good	0.9	1.00 (0.65–1.55)			0.5	0.85 (0.51–1.43)			0.09	1.46 (0.94–2.27)	**0.01**	1.84 (1.13–2.99)
Moderate/difficult to tell	0.8	0.94 (0.61–1.45)			0.3	0.76 (0.46–1.27)			**0.03**	1.65 (1.06–2.56)	**0.01**	1.81 (1.14–2.87)
Rather bad, bad or very good		Reference				Reference				Reference		Reference
**Variable**	**Too Little Dietary Fiber Intake**				**Too Little Vitamin Intake**				**Too Little Consumption of Vegetables and Fruits**			
	**Simple Logistic Regression**		**Multivariable** **Logistic Regression**		**Simple Logistic Regression**		**Multivariable** **Logistic** **Regression**		**Simple Logistic Regression**		**Multivariable** **Logistic Regression**	
	***p***	**OR (95%CI)**	***p***	**OR (95%CI)**	***p***	**OR (95%CI)**	***p***	**OR (95%CI)**	***p***	**OR (95%CI)**	***p***	**OR (95%CI)**
Gender												
Female	**0.01**	1.38 (1.07–1.76)	**0.01**	1.38 (1.07–1.78)	0.07	1.25 (0.98–1.59)			**0.01**	1.39 (1.09–1.78)	**0.02**	1.38 (1.05–1.79)
Male		Reference		Reference		Reference				Reference		Reference
Age (years)												
18–34		Reference		Reference	**0.03**	1.54 (1.05–2.26)	0.1	1.41 (0.91–2.19)		Reference		Reference
35–49	0.5	1.13 (0.82–1.57)	0.6	1.09 (0.79–1.52)	0.3	1.25 (0.84–1.85)	0.2	1.22 (0.80–1.84)	0.08	1.33 (0.97–1.83)	0.2	1.28 (0.90–1.82)
50–64	0.2	1.22 (0.88–1.69)	0.6	1.11 (0.79–1.56)	0.5	0.87 (0.59–1.30)	0.7	0.90 (0.60–1.35)	**0.001**	1.71 (1.23–2.38)	0.07	1.43 (0.98–2.10)
65+	0.03	1.55 (1.05–2.27)	0.2	1.35 (0.90–2.03)		Reference		Reference	**<0.001**	2.08 (1.38–3.12)	0.03	1.68 (1.05–2.69)
Having higher education												
Yes	**<0.001**	1.75 (1.37–2.25)	**<0.001**	1.78 (1.38–2.29)	**<0.001**	1.66 (1.30–2.12)	**<0.001**	1.54 (1.20–1.99)	**0.005**	1.44 (1.12–1.85)	**0.02**	1.36 (1.04–1.78)
No		Reference		Reference		Reference		Reference		Reference		Reference
Ever married												
Yes	0.3	0.88 (0.69–1.14)			**0.003**	Reference	0.1	Reference	0.3	1.16 (0.90–1.50)		
No		Reference				1.45 (1.13–1.86)		1.24 (0.93–1.66)		Reference		
Having children												
Yes	0.2	1.18 (0.91–1.53)			0.2	0.84 (0.65–1.08)			**0.007**	1.42 (1.10–1.83)	0.7	1.07 (0.79–1.44)
No		Reference				Reference				Reference		Reference
Number of household members												
Living alone	0.6	0.91 (0.64–1.31)			0.1	Reference			0.6	1.10 (0.77–1.58)		
2 or more		Reference				1.30 (0.91–1.85)				Reference		
Place of residence												
Rural		Reference				Reference		Reference		Reference		Reference
City below 20,000 residents	0.3	0.81 (0.53–1.23)			0.7	0.93 (0.62–1.39)	0.7	0.93 (0.62–1.40)	0.6	1.11 (0.74–1.67)	0.6	1.10 (0.73–1.67)
City from 20,000 to 99,999 residents	0.2	1.26 (0.90–1.78)			0.06	1.38 (0.99–1.93)	0.05	1.41 (1.00–1.99)	**0.02**	1.50 (1.06–2.13)	0.2	1.29 (0.90–1.85)
City from 100,000 to 499,999 residents	0.7	1.06 (0.74–1.52)			0.1	1.32 (0.93–1.86)	0.2	1.30 (0.91–1.85)	**0.03**	1.49 (1.04–2.14)	0.08	1.39 (0.96–2.01)
City above 500,000 residents	0.1	1.37 (0.93–2.02)			**0.006**	1.71 (1.16–2.51)	**0.02**	1.63 (1.09–2.44)	0.08	1.43 (0.96–2.12)	0.4	1.20 (0.80–1.82)
Occupational status												
Active	0.8	1.03 (0.80–1.33)			0.05	1.28 (0.99–1.64)			0.1	0.82 (0.63–1.06)		
Passive		Reference				Reference				Reference		
Self-reported financial status												
Rather good, good or very good	0.4	1.17 (0.84–1.63)			0.3	1.19 (0.86–1.64)			**0.04**	1.40 (1.01–1.95)	**0.01**	1.56 (1.10–2.20)
Moderate/difficult to tell	0.5	1.13 (0.81–1.57)			0.2	1.24 (0.90–1.71)			**0.03**	1.45 (1.04–2.01)	**0.01**	1.55 (1.10–2.17)
Rather bad, bad or very good		Reference				Reference				Reference		Reference
Presence of chronic diseases												
Yes	**0.005**	1.43 (1.11–1.83)	**0.02**	1.37 (1.05–1.79)	0.6	1.06 (0.84–1.35)			**<0.001**	1.70 (1.32–2.19)	**0.002**	1.56 (1.18–2.06)
No		Reference		Reference		Reference				Reference		Reference
Self-reported health status												
Rather good, good or very good	0.8	1.07 (0.68–1.67)			**0.03**	1.65 (1.05–2.58)	0.09	1.49 (0.94–2.37)	0.6	1.14 (0.72–1.80)		
Moderate/difficult to tell	0.8	0.95 (0.60–1.48)			0.4	1.23 (0.79–1.93)	0.3	1.25 (0.79–1.98)	0.4	0.82 (0.53–1.30)		
Rather bad, bad or very good		Reference				Reference		Reference		Reference		
**Variable**	**Too Little Intake of Calcium and Magnesium**						**Too Little Consumption of Fish and Oils**					
	**Simple Logistic Regression**			**Multivariable Logistic Regression**			**Simple Logistic Regression**			**Multivariable Logistic Regression**		
	***p***	**OR (95%CI)**		***p***	**OR (95%CI)**		***p***	**OR (95%CI)**		***p***	**OR (95%CI)**	
Gender												
Female	0.3	1.15 (0.90–1.47)					**0.01**	1.36 (1.07–1.74)		**0.02**	1.36 (1.06–1.74)	
Male		Reference						Reference			Reference	
Age (years)												
18–34	0.9	1.02 (0.69–1.49)						Reference			Reference	
35–49	0.6	0.91 (0.61–1.35)					**0.03**	1.44 (1.05–1.98)		**0.04**	1.41 (1.02–1.95)	
50–64	0.6	0.91 (0.61–1.35)					**0.02**	1.47 (1.07–2.02)		0.2	1.25 (0.89–1.75)	
65+		Reference					**0.004**	1.76 (1.20–2.58)		0.07	1.47 (0.98–2.20)	
Having higher education												
Yes	**0.004**	1.43 (1.12–1.83)		**0.004**	1.43 (1.12–1.83)		**0.004**	1.44 (1.13–1.84)		**0.007**	1.42 (1.10–1.82)	
No		Reference			Reference			Reference			Reference	
Ever married												
Yes	0.5	0.91 (0.71–1.17)					0.5	1.08 (0.84–1.39)				
No		Reference						Reference				
Having children												
Yes	0.6	0.94 (0.73–1.21)					0.06	1.27 (0.99–1.63)				
No		Reference						Reference				
Number of household members												
Living alone	0.1	0.75 (0.52–1.08)					0.7	0.93 (0.66–1.33)				
2 or more		Reference						Reference				
Place of residence												
Rural		Reference						Reference			Reference	
City below 20,000 residents	0.3	0.79 (0.52–1.20)					0.6	0.90 (0.60–1.36)		0.6	0.88 (0.58–1.34)	
City from 20,000 to 99,999 residents	0.2	1.23 (0.88–1.72)					0.06	1.39 (0.99–1.95)		0.2	1.23 (0.87–1.74)	
City from 100,000 to 499,999 residents	0.4	1.15 (0.81–1.64)					**0.04**	1.44 (1.02–2.05)		0.08	1.37 (0.96–1.95)	
City above 500,000 residents	0.2	1.30 (0.88–1.91)					0.1	1.36 (0.92–2.00)		0.4	1.17 (0.79–1.74)	
Occupational status												
Active	0.06	1.28 (0.99–1.65)					0.5	0.91 (0.71–1.17)				
Passive		Reference						Reference				
Self-reported financial status												
Rather good, good or very good	0.2	1.26 (0.90–1.75)					0.3	0.84 (0.61–1.17)				
Moderate/difficult to tell	0.3	1.17 (0.84–1.63)					0.8	0.96 (0.70–1.33)				
Rather bad, bad or very good		Reference						Reference				
Presence of chronic diseases												
Yes	0.1	1.22 (0.96–1.56)					**<0.001**	1.59 (1.24–2.03)		**0.004**	1.48 (1.14–1.93)	
No		Reference						Reference			Reference	
Self-reported health status												
Rather good, good or very good	0.3	1.27 (0.81–2.01)					0.3	1.30 (0.83–2.04)				
Moderate/difficult to tell	0.3	1.27 (0.81–2.00)					0.4	1.22 (0.78–1.91)				
Rather bad, bad or very good		Reference						Reference				

## Data Availability

Data are available on reasonable request. The dataset used to conduct the analyses is available from corresponding author on reasonable request.
